# Psychodidae (Diptera) of Azerbaijan and Georgia – faunistics with biodiversity notes

**DOI:** 10.3897/zookeys.1049.66063

**Published:** 2021-06-15

**Authors:** Jan Ježek, Peter Manko, Jozef Oboňa

**Affiliations:** 1 Department of Entomology, National Museum, Cirkusová 1740, CZ – 193 00 Praha 9 – Horní Počernice, Czech Republic; 2 Department of Ecology, Faculty of Humanities and Natural Sciences, University of Prešov, 17. novembra 1, SK – 081 16 Prešov, Slovakia

**Keywords:** Biodiversity, Caucasus, faunistics, moth flies, new records, new synonymy, taxonomy, zoogeography

## Abstract

Records of 46 Psychodidae (Sycoracinae 1, Trichomyiinae 1, Psychodinae 44) species/subspecies are presented in this paper based on specimens collected by sweep-netting in Azerbaijan and Georgia in 2019. Nine species are recorded for the first time since their original description; 12 species are new for Transcaucasia; 22 species are new for Azerbaijan; and 17 species are new for Georgia. *Saraiella ressli montana* Ježek, 1990 is proposed as a synonym of *S. ressli* Wagner, 1983, **syn. nov.** Knowledge of some aspects of the ecology and biogeography of selected (especially rare) species has been expanded and a clear pattern was found in species richness, rare species, and new records in relation to land use, habitat diversity, and preservation of the environment surrounding the sampling site.

## Introduction

The purpose of faunistic studies is the registration of species. Intensive faunistic studies are necessary if we want to determine species richness on a local scale and track long‐term changes in species diversity (Ejsmont‐Karabin 2019). This applies in particular to areas that are of the utmost importance from the point of view of biodiversity but are still insufficiently researched for some groups of organisms. One such biodiversity hotspot is the Caucasus, and one of its hitherto insufficiently known groups is the family Psychodidae. For the above reasons, this is another contribution to knowledge of the Diptera: Psychodidae, especially Sycoracinae, Trichomyiinae and Psychodinae from Azerbaijan and Georgia.

Psychodidae is a relatively species-rich family, with nearly 500 species known to occur in Europe and the adjacent island areas; for more details, see Wagner (2018). The following authors contributed considerably to knowledge on the Transcaucasian Psychodidae: Perfiľev (1966); Lewis (1982); Artemiev and Neronov (1984); Vaillant (1972–1983); Vaillant and Joost (1983); Wagner (1981, 1990, 2018); Wagner and Joost (1986, 1988); Gugushvili and Lomtadze (2002); Wagner et al. (2002); Ježek (1987, 2004, 2006a, 2007, 2009); Melaun et al. (2014); Ježek et al. (2014, 2018) and recently Oboňa et al. (2017, 2019a).

**Figure 1.**
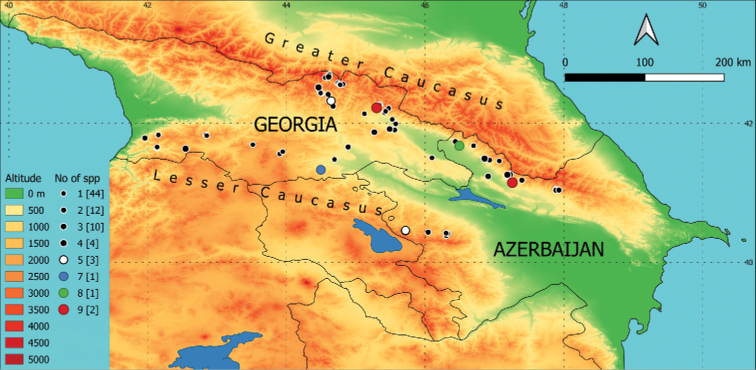
Recent collecting sites on a map of the area of interest. Different colours (altitude) indicate the elevation in m a.s.l., and circle diameters and colours indicate the number of species recorded at each sampling site. The number of sites where the specific number of species is present is given in brackets.

The Caucasus consists of several mountain ranges with different connectivity and serves heterogeneous environments, with different climatic conditions between lowlands and highlands as a result of orogenic activity in the Miocene-Pliocene (Hrivniak et al. 2020). The parts of Georgia and Azerbaijan sampled in this research cover parts of the Greater Caucasus and Lesser Caucasus (see Fig. 1), separated by the Rieni and Kura river valleys and partly connected in the Likhi (Surami) mountain range at a relatively high elevation, with the lowest point of 949 m a.s.l. (Surami Pass).

Following our results and findings in Transcaucasia (Ježek et al. 2018, 2021; Oboňa et al. 2017, 2019a), which indicate the relatively high diversity of this family in the Caucasus, we continued to collect material in 2019. We did so in an effort to better understand the diversity and occurrence of the family Psychodidae in the Caucasus and to make the data on the faunistics and biodiversity of this group more complete.

## Materials and methods

Dipterans were collected by PM, JO, T. Kovács (Mátra Museum of Hungarian Natural History Museum, Gyöngyös, Hungary), D. Murányi (Eszterházy Károly University, Eger, Hungary), and G. Vinçon (Grenoble, France) in the three periods between iv–v, vii, and ix–x, all in 2019, by sweep-netting from vegetation growing along watercourses and lakes. The captured specimens were preserved in 75% ethanol in the field. In the laboratory, specimens of Psychodidae were cleared in chloralphenol, treated in xylol, and mounted on glass slides in Canada balsam, subsequently identified by JJ and deposited at the National Museum (Natural History Museum), Department of Entomology, Prague, Czech Republic. The slides are labelled with the inventory slide number of the family Psychodidae (Inv. No.) and are included in the Diptera collection of National Museum Prague collections (**NMPC**), see Tkoč et al. (2014). The nomenclature is modified from Vaillant (1972–1983) and Wagner (1990, 2018) using the classifications of Ježek (1990a, 2007); Ježek and van Harten (2005); Omelkova and Ježek (2012); Oboňa and Ježek (2014) and Kroča and Ježek (2015, 2019). We use the term “Transcaucasia” (Armenia, Azerbaijan, and Georgia) according to the Catalogue of Palaearctic Diptera; for more details, see Wagner (1990). Information on the distribution (simple distribution overview) is given for the species recorded from Azerbaijan and Georgia for the first time.

In the list of localities in Suppl. material 1: Table S1, data are presented in the following order: number (in brackets), territorial unit (district, region etc.), name of the locality, nearest settlement (where appropriate), specified location and habitat, coordinates, and elevation. The localities are listed alphabetically. Data for the material examined are arranged in the following order: number, date of collection, number of males and females, slide number and collector’s or determinator’s name (abbreviated).

Abbreviations used:

**TK** Tibor Kovács;

**DM** Dávid Murányi;

**GV** Gilles Vinçon.

**Figure 2.**
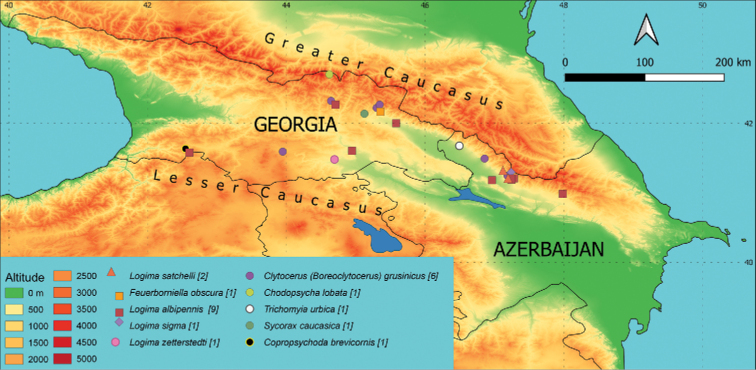
Occurrence of individual species on a map of the area of interest. Different colours (altitude) indicate the elevation in m a.s.l. and different symbols mark the sampling sites with occurrence of the species. When species co-occurred at the locality, an offset is shown in the legend and described in the Materials and methods. The number of sites where the species is present is given in brackets.

**Figure 3.**
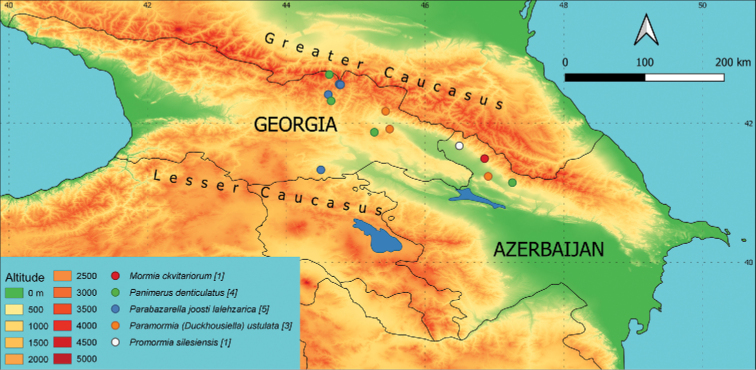
Occurrence of individual species on a map of the area of interest. Different colours (altitude) indicate the elevation in m a.s.l. and different symbols mark the sampling sites with occurrence of the species. The number of sites where the species is present is given in brackets.

**Figure 4.**
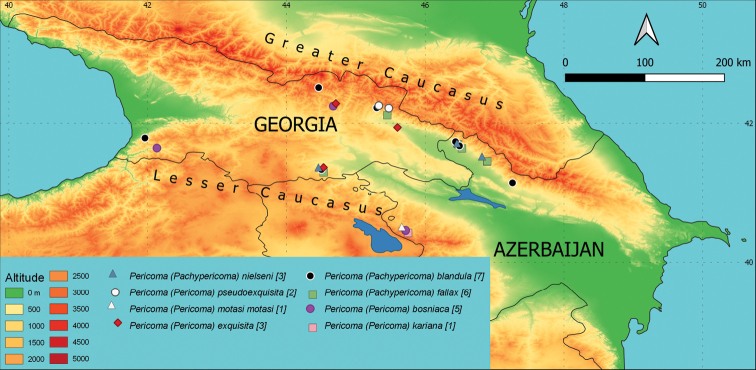
Occurrence of individual species on a map of the area of interest. Different colours (altitude) indicate the elevation in m a.s.l. and different symbols the sampling sites with occurrence of the species. When species co-occurred at a locality, an offset is shown in the legend and described in the Materials and methods. The number of sites where the species is present is given in brackets.

**Figure 5.**
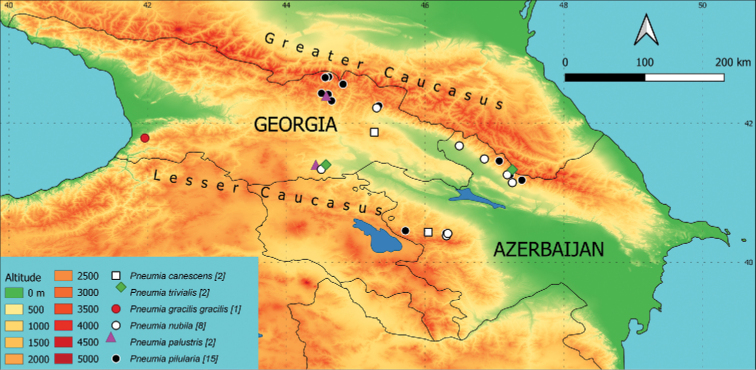
Occurrence of individual species on a map of the area of interest. Different colours (altitude) indicate the elevation in m a.s.l. and different symbols the sampling sites with occurrence of the species. When the species co-occurred at the locality, an offset is shown in the legend and described in the Materials and methods. The number of sites where the species is present is given in brackets.

**Figure 6.**
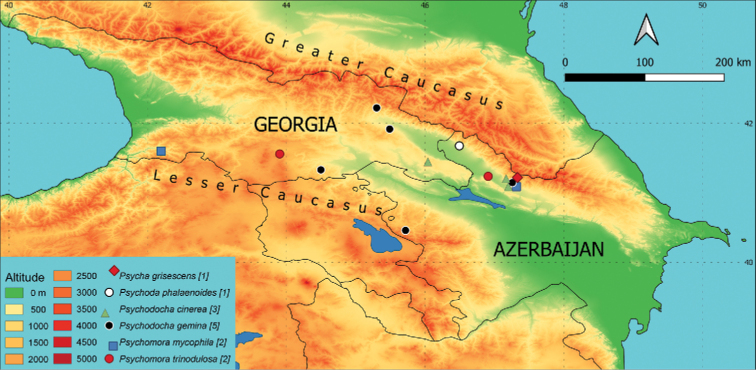
Occurrence of individual species on a map of the area of interest. Different colours (altitude) indicate the elevation in m a.s.l. and different symbols the sampling sites with occurrence of species. When species co-occurred at the locality, an offset is shown in the legend and described in the Materials and methods. The number of sites where the species is present is given in brackets.

**Figure 7.**
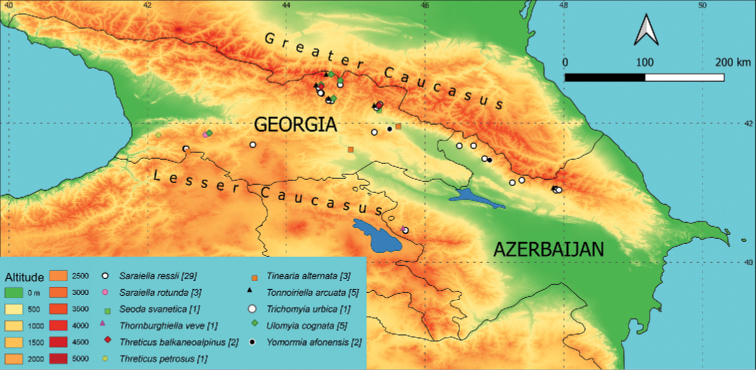
Occurrence of individual species on a map of the area of interest. Different colours (altitude) indicate the elevation in m a.s.l. and different symbols the sampling sites with occurrence of the species. The number of sites where the species is present is given in brackets after the species name. When species co-occurred at the locality, an offset is also shown in the legend and described in the Materials and methods.

The distribution of the sampling sites (Fig. 1) and species recorded in this study (Figs 2–7) are presented in maps prepared using data derived from USGS/NASA SRTM data providing seamless continuous topography surfaces (Jarvis et al. 2008). Areas with different elevations were painted in QGIS (version: 3.10.10-A Coruña) with the ‘Band Rendering’ (Singleband Pseudocolor, Oranges Colour Ramp, the colour for values from > 0 to ≤ 500 was changed to green). When species co-occurred at the locality, the offset was set to x = 1 mm, y = 1 mm for species marked with square symbol, x = -1 mm, y = -1 mm for species marked with triangles and x = 1 mm, y = -1 mm for diamonds, respectively.

## Results

We analysed material obtained in 2019 from 80 sites located in Transcaucasia in the territories of Azerbaijan and Georgia. Based on 182 slides from this material, we identified 46 species/subspecies belonging to three subfamilies. Below we present a list of recorded species and their distribution, together with the information on the material examined and notes.

### Family Psychodidae

#### Subfamily Sycoracinae

##### Sycorax caucasica

Taxon classificationAnimaliaDipteraPsychodidae

Ježek, 1990

7AA20BE6-35DF-59B2-AF12-BD3FF0DC5C9C

###### Material examined.

**Georgia**: G 13, 8.7.2019 (Fig. 2), 1♂, slide Inv. No. 25630, leg. PM, GV.

###### Distribution.

Caucasian species, not rare in Abkhazia (Ježek 1990a).

###### Note.

Species known only from the original description from Abkhazia (Ježek 1990a). First record since the original description, first record from Georgia, from a territory other than Abkhazia. Although this is a typical mountain species (elevation of ca. 2000 m a.s.l.), it is also known from the small hills (Ježek 1990a) and foothills of the Greater Caucasus (see above).

#### Subfamily Trichomyiinae

##### Trichomyia urbica

Taxon classificationAnimaliaDipteraPsychodidae

Haliday in Curtis, 1839

C1A8651D-80BF-55CA-BA3D-AAF352278776

###### Material examined.

**Azerbaijan**: A 02, 9.5.2019, 1♂, slide Inv. No. 25663, leg. JO (Fig. 2).

###### Distribution.

Rare species, known from Austria, Belgium, Czech Republic, Denmark, France, Germany, Great Britain, Greece, Hungary, Ireland, the Netherlands, Norway, Poland, Romania, Slovakia, and Sweden; larvae are xylophagous and occur in shaded slope spring areas and some other habitats with decaying organic matter (Ježek 2003; Ježek and Omelková 2012).

###### Note.

First record for Azerbaijan and Transcaucasia.

#### Subfamily Psychodinae

##### Clytocerus (Boreoclytocerus) grusinicus

Taxon classificationAnimaliaDipteraPsychodidae

Wagner, 1981

9B97C88B-D721-5A32-B30A-C402B053449B

###### Material examined.

**Azerbaijan**: A 14, 7.5.2019, 1♂, slide Inv. No. 25708, leg. JO; A 22, 5.5.2019, 1♂, slide Inv. No. 25625, leg. JO. **Georgia**: G 09, 2.5.2019, 2♂♂, slide Inv. No. 25642 and 25727, leg. JO; G 14, 2.5.2019, 1♂, slide Inv. No. 25710, leg. JO; G 26, 15.7.2019, 1♂, slide Inv. No. 25583, leg. TK, DM, GV; G 40, 13.7.2019, 1♂, slide Inv. No. 25633, leg. TK, PM, DM, GV (Fig. 2).

###### Distribution.

Rare Caucasian species known only from a few localities (Wagner 1981; Oboňa et al. 2019).

###### Note.

Described from Georgia by Wagner (1981) on the basis of the only holotype from a right tributary above the village Kwarchi. After the second record from Georgia (Oboňa et al. 2019), the species was recorded from another six sites in this study. Species found in low numbers in a range of elevations from 295 m (Oboňa et al. 2019) to 1520 m a.s.l. (see above). First records for Azerbaijan.

##### Copropsychoda brevicornis

Taxon classificationAnimaliaDipteraPsychodidae

(Tonnoir, 1940)

DA18CB24-53D4-50A0-87AB-63238E0659C6

###### Material examined.

**Georgia**: G 03, 27.9.2019, 1♀, slide Inv. No. 25604, leg. PM (Fig. 2).

###### Distribution.

Coprophagous species with a Western Palaearctic distribution. Known from Europe (Belgium, Great Britain, France, Germany, Ireland, the Netherlands, and Norway) (Ježek et al. 2018b; Wagner 2018).

###### Note.

First record for Georgia and Transcaucasia.

##### Feuerborniella obscura

Taxon classificationAnimaliaDipteraPsychodidae

(Tonnoir, 1919)

15A297F7-E9A2-5D54-85A7-4AA0017F5F02

###### Material examined.

**Georgia**: G 09, 2.5.2019, 1♀, slide Inv. No. 25731, leg JO (Fig. 2).

###### Distribution.

A common European species, distributed in Central Europe, along the Atlantic coast, in the British Isles and reaching the Apennines and the Balkans in the south, known from habitats with sprayed moss cushions, spring areas, and stream meanders across a wide range of elevations (Ježek and Omelková 2012; Oboňa et al. 2019b).

###### Note.

First record for Georgia and Transcaucasia.

##### Chodopsycha lobata

Taxon classificationAnimaliaDipteraPsychodidae

(Tonnoir, 1940)

630802C1-AFD3-5D09-85D4-8E4C3F4F694D

###### Material examined.

**Georgia**: G 28, 6.7.2019, 1♂, slide Inv. No. 25609, leg. PM (Fig. 2).

###### Distribution.

Generally common species, occurring across Europe to Transcaucasia, associated with fungi (Kroča and Ježek 2019).

###### Note.

First record for Georgia, from a territory other than Abkhazia.

##### Logima albipennis

Taxon classificationAnimaliaDipteraPsychodidae

(Zetterstedt, 1850)

8855005F-707E-5CEF-88B8-0644C89DAB75

###### Material examined.

**Azerbaijan**: A 9, 6.5.2019, 1♀, slide Inv. No. 25716, leg. JO; A 18, 10.5.2019, 1♀, slide Inv. No. 25644, leg. JO; A 20, 5.5.2019, 1♀, slide Inv. No. 25720, leg. JO; A 21, 8.5.2019, 1♀, slide Inv. No. 25752, leg. JO; A 22, 5.5.2019, 1♀, slide Inv. No. 25627, leg. JO; A 23, 6.5.2019, 2♀♀, slide Inv. No. 25688 and 25695, leg. JO. **Georgia**: G 03, 27.9.2019, 1♀, slide Inv. No. 25605, leg. PM; G 18, 1.5.2019, 1♀, slide Inv. No. 25715, leg. JO; G 49, 28.4.2019, 1♀, slide Inv. No. 25670, leg. JO; G 56, 26.4.2019, 1♀, slide Inv. No. 25696, leg. JO (Fig. 2).

###### Distribution.

A cosmopolitan species known from Europe (Austria, Azores, Belgium, Bosnia and Herzegovina, Bulgaria, Czech Republic, Denmark, Finland, France, Germany, Great Britain, Greece, Hungary, Ireland, Italy, Luxemburg, Madeira, the Netherlands, Norway, Poland, Portugal, Romania, Russia, Sardinia, Serbia, Slovakia, Slovenia, and Sweden). In Asia from Armenia, Azerbaijan, Afghanistan, China, India, Japan, North Korea, Syria, and Turkey. In Africa, from Algeria, the Canary Islands, Gambia, South Africa, and Tunisia. Also from Australia, New Zealand, South America, USA, Campbell Island, Juan Fernandez Islands, Kerguelen Island, and Macquarie Island (Ježek and Yağci 2005; Kvifte 2012; Afzan and Belqat 2016; Ježek et al. 2018).

###### Note.

First record for Georgia.

##### Logima satchelli

Taxon classificationAnimaliaDipteraPsychodidae

(Quate, 1955)

C444E378-73CF-593F-B05D-53A81ABC431E

###### Material examined.

**Azerbaijan**: A 21, 8.5.2019, 1♀, slide Inv. No. 25751, leg. JO; A 23, 6.5.2019, 1♀, slide Inv. No. 25689, leg. JO (Fig. 2).

###### Distribution.

Common Holarctic species, eurybiotic (Kroča and Ježek 2019).

##### Logima sigma

Taxon classificationAnimaliaDipteraPsychodidae

(Kincaid, 1899)

3850957D-26CC-515C-8B9F-8E04D7F6B511

###### Material examined.

**Azerbaijan**: A 22, 5.5.2019, 1♀, slide Inv. No. 25626, leg. JO (Fig. 2).

###### Distribution.

Uncommon Holarctic species. Recorded from Austria, Belgium, Czech Republic, France, Great Britain, Norway, Poland, Slovakia, Spain (incl. Madeira); Antipodes Is., Auckland L. (lake or lakes), Australia, Campbell L., Chile, Enderby L., Ewing L., French L., Macquarie L., New Zealand, Ocean L., Rose L., Saint Helena, and USA (Andersen and Håland 1995; Ježek 2003; Kvifte et al. 2011; Kvifte and Andersen 2012; Elgueta and Ježek 2014; Haselboeck 2016; Ježek et al. 2018b; Oboňa and Kozánek 2018; Wagner 2018).

###### Note.

First record for Azerbaijan and Transcaucasia.

##### Logima zetterstedti

Taxon classificationAnimaliaDipteraPsychodidae

Ježek, 1983

F337EEE0-C9DA-5544-9894-FE37F64B4FCA

###### Material examined.

**Georgia**: G 23, 29.4.2019, 1♀, slide Inv. No. 25714, leg. JO (Fig. 2).

###### Distribution.

Common European and Western Siberian species recorded from Belgium, Czech Republic, Greit Britain, Slovakia, Slovenia, and the Netherlends (Omelková and Ježek 2012; Ježek et al. 2018b; Wagner 2018).

###### Note.

First record for Georgia and Transcaucasia.

##### Mormia ckvitariorum

Taxon classificationAnimaliaDipteraPsychodidae

Ježek, 1987

40372BF0-BB2A-512C-988B-FEC58A759490

###### Material examined.

**Azerbaijan**: A 14, 7.5.2019, 3♂♂, slide Inv. No. 25738, 25739 and 25740, leg. JO (Fig. 3).

###### Note.

Species known only from the original description from Abkhazia (Ježek 1987). First record since the original description, first record for Azerbaijan. Species known only from lowland and foothills of the Greater Caucasus.

##### Promormia silesiensis

Taxon classificationAnimaliaDipteraPsychodidae

(Ježek, 1983)

77159C59-3563-5287-BE24-A1C5DCBF8AB1

###### Material examined.

**Azerbaijan**: A 02, 9.5.2019, 1♂, slide Inv. No. 25661, leg. JO (Fig. 3).

###### Distribution.

Species known only from Czech Republic, Greece, Slovakia, and Slovenia; until now considered as a rare European species (Ježek 1983; Omelková and Ježek 2012; Oboňa and Ježek 2014).

###### Note.

Based on the record from the southern slope of the Greater Caucasus foothills (see above), it is necessary to reconsider it as a European and Transcaucasian species. First record for Azerbaijan and Transcaucasia.

##### Panimerus denticulatus

Taxon classificationAnimaliaDipteraPsychodidae

Krek, 1971

19F78687-6D71-5F77-9269-E82EE27E749A

###### Material examined.

**Azerbaijan**: A 23, 6.5.2019, 1♂, slide Inv. No. 25693, leg. JO (Fig. 3). **Georgia**: G 12, 9.7.2019, 1♂, slide Inv. No. 25613, leg. PM, GV; G 32, 2.7.2019, 1♂, slide Inv. No. 25615, leg. TK, DM, GV; G 40, 13.7.2019, 1♂, slide Inv. No. 25634, leg. TK, PM, DM, GV.

###### Distribution.

Locally common species, known from several European countries: Belgium, Austria, Great Britain, Ireland, Bosnia and Herzegovina, Czech Republic, Greece, and Macedonia (Omelková and Ježek 2012; Ježek et al. 2018b).

###### Note.

Records scattered over a large area of Transcaucasia (Fig. 3) suggest that the species is also relatively common in Transcaucasia. It is necessary to reconsider it as a European and Transcaucasian species. First record for Azerbaijan, Georgia, and Transcaucasia.

##### Paramormia (Duckhousiella) ustulata

Taxon classificationAnimaliaDipteraPsychodidae

(Walker, 1856)

45B28BBF-0AE6-5334-9620-4CC8CDB87B4D

###### Material examined.

**Azerbaijan**: A 19, 7.5.2019, 1♂, 1♀, slide Inv. No. 25671 and 25723, leg. JO. **Georgia**: G 19, 30.4.2019, 1♂, slide Inv. No. 25649, leg. JO; G 21, 3.5.2019, 1♀, slide Inv. No. 25681, leg. JO (Fig. 3).

###### Distribution.

Holarctic species, known from most of Europe, very common locally, mainly in extreme localities (salt works, thermal springs, calcareous water, mineral-rich springs). Also recorded in Algeria, Morocco, Israel, Armenia, Azerbaijan, Afghanistan, Iran, and USA (Ježek and Yağci 2005; Salmela et al. 2014; Kvifte et al. 2016; Ježek et al. 2018a; Oboňa et al. 2019a).

###### Note.

First record for Georgia.

##### Parabazarella joosti lalehzarica

Taxon classificationAnimaliaDipteraPsychodidae

Ježek, 1990

8C1AF31E-2EBE-5644-A711-C9D49CE4B58F

###### Material examined.

**Georgia**: G 24, 29.4.2019, 3♂♂, slide Inv. No. 25678, 25734 and 25745, leg. JO; G 35, 11.7.2019, 1♂, slide Inv. No. 25616, leg. TK, PM, DM; G 34, 11.7.2019, 1♂, slide Inv. No. 25581, leg. TK, PM, DM; G 35, 11.7.2019, 1♂, slide Inv. No. 25571, leg. TK, PM, DM, GV; G 43, 11.7.2019, 1♂, slide Inv. No. 25617, leg. TK, PM, DM, GV (Fig. 3).

###### Distribution.

*Bazarella joosti* Vaillant, 1983, was described from Schelda nr. Kashadl (Central Caucasus: Georgia, probably Gora Shkhel’da, omitted (overlooked) from Georgia in Oboňa et al. 2019a); the subspecies lalehzrica is known only from the original description (Ježek 1990b) from S. E. Iran, Kerman province, Kuh-e Lalehzar (top m a.s.l. 4374, 3850–4374 m), holotype and 9 paratypes (males).

###### Note.

*Parabazarella* Vaillant, 1983 was raised to genus level by Ježek (2001) after previously being considered a subgenus of *Bazarella* Vaillant, 1961. This is the first record since the original description.

##### Pericoma (Pachypericoma) blandula

Taxon classificationAnimaliaDipteraPsychodidae

Eaton, 1893

87AA84FF-7E35-572E-B569-1909E76C8C4B

###### Material examined.

**Azerbaijan**: A 01 9.5.2019, 1♂, slide Inv. No. 25624, leg. JO; A 02, 9.5.2019, 1♂, slide Inv. No. 25665, leg. JO; A 23, 6.5.2019, 1♂, slide Inv. No. 25687, leg. JO. **Georgia**: G 02, 24.9.2019, 1♂, slide Inv. No. 25584, leg. TK, PM; G 09, 2.5.2019, 1♂, slide Inv. No. 25732, leg. JO; G 25, 29.4.2019, 1♂, slide Inv. No. 25713, leg. JO; G 47, 9.7.2019, 1♂, slide Inv. No. 25638, leg. TK, PM, DM, GV.

###### Distribution.

Common European and Transcaucasian species, penetrates into North Africa (Kroča and Ježek 2019).

##### Pericoma (Pachypericoma) fallax

Taxon classificationAnimaliaDipteraPsychodidae

Eaton, 1893

F0520A8E-B010-50D2-BF67-C42C139FF9AA

###### Material examined.

**Azerbaijan**: A 01, 9.5.2019, 1♂, slide Inv. No. 25707, leg. JO; A 02, 9.5.2019, 1♂, slide Inv. No. 25664, leg. JO; A 13, 7.5.2019, 1♂, slide Inv. No. 25743, leg. JO; A 14, 7.5.2019, 1♂, slide Inv. No. 25709, leg. JO. **Georgia**: G 20, 3.5.2019, 1♂, slide Inv. No. 25717, leg. JO; G 24, 29.4.2019, 1♂, slide Inv. No. 25749, leg. JO.

###### Distribution.

European, western Siberian, and Caucasian species (Kroča and Ježek 2019).

###### Note.

First record for Georgia, from a territory other than Abkhazia.

##### Pericoma (Pachypericoma) nielseni

Taxon classificationAnimaliaDipteraPsychodidae

Kvifte, 2010

2991DEDC-BB5F-5286-8608-8836F1FD74D2

###### Material examined.

**Azerbaijan**: A 02, 9.5.2019, 1♂, slide Inv. No. 25662, leg. JO; A 13, 7.5.2019, 1♂, slide Inv. No. 25742, leg. JO. **Georgia**: G 24, 29.4.2019, 1♂, slide Inv. No. 25747, leg. JO.

###### Distribution.

Not a common European species, known from Czech Republic, Denmark, Finland, France, Norway, Slovakia, and Ukraine (Ježek 2009; Kvifte 2010; Kvifte et al. 2011; Ježek et al. 2017).

###### Note.

It is necessary to reconsider this as a European and Transcaucasian species. First record for Azerbaijan, Georgia, and Transcaucasia.

##### Pericoma (Pericoma) bosniaca

Taxon classificationAnimaliaDipteraPsychodidae

Krek, 1966

18CAD882-D14F-5203-B938-E470F7F638AC

###### Material examined.

**Azerbaijan**: A 04, 1.10.2019, 1♂, slide Inv. No. 25597, leg. PM; A 05, 1.10.2019, 1♂, slide Inv. No. 25578, leg. PM; A 06, 1.10.2019, 1♂, slide Inv. No. 25574, leg. PM. **Georgia**: G 01, 25.9.2019, 1♂, slide Inv. No. 25601, leg. PM; G 41, 27.4.2019, 2♂♂, slides Inv. No. 25653 and 25654, leg. JO.

###### Distribution.

Species known from Bosnia and Herzegovina, Bulgaria, Montenegro, Serbia, Macedonia, Armenia, and Azerbaijan (Krek 1966, 1967a, 1967b, 1970, 1972, 1974, 1979a, 1999; Krek et al. 1976; Mučibabič et al. 1984; Vaillant 1978; Wagner and Joost 1988; Wagner 1990; Ježek et al. 2018; Oboňa et al. 2019a).

###### Note.

First record for Georgia.

##### Pericoma (Pericoma) exquisita

Taxon classificationAnimaliaDipteraPsychodidae

Eaton, 1893

FB7404DD-E6AA-5EDB-A5CE-74DA43B7F99A

###### Material examined.

**Georgia**: G 17, 30.4.2019, 1♂, slide Inv. No. 25657, leg. JO; G 24, 29.4.2019, 2♂♂, slide Inv. No. 25736 and 25746, leg. JO; G 41, 27.4.2019, 1♂, slide Inv. No. 25652, leg. JO.

###### Distribution.

Species widespread in Europe, North Africa, and Transcaucasia (Armenia, Azerbaijan). In Europe, known from Albania, Austria, Belgium, Bosnia and Herzegovina, Bulgaria, Czech Republic, Crete, Croatia, France, Germany, Great Britain, Greece, Hungary, Ireland, Italy, Macedonia, Montenegro, Poland, Serbia, Slovakia, Slovenia, Spain, and Ukraine (Ježek 2004, 2009; Kvifte et al. 2013; Wagner 2013; Oboňa and Ježek 2014; Ježek et al. 2017; Oboňa et al. 2019a).

###### Note.

First record for Georgia.

##### Pericoma (Pericoma) kariana

Taxon classificationAnimaliaDipteraPsychodidae

Vaillant, 1978

A85D87E2-E7FA-5428-B9F6-162AA66DAC46

###### Material examined.

**Azerbaijan**: A 05, 1.10.2019, 1♂, slide Inv. No. 25577, leg. PM.

###### Distribution.

Species known only from the original description from Greece (Vaillant 1978).

###### Note.

Extremely rare species. First record since the original description, and therefore a first record for Azerbaijan.

##### Pericoma (Pericoma) motasimotasi

Taxon classificationAnimaliaDipteraPsychodidae

Vaillant, 1978

617E1834-269C-5EE8-A848-A6E3985BB567

###### Material examined.

**Azerbaijan**: A 04, 1.10.2019, 1♂, slide Inv. No. 25596, leg. PM.

**Distribution.** An extremely rare species known from Bulgaria, Georgia, Greece, Macedonia, Serbia, and Romania (Wagner and Joost 1988; Oboňa et al. 2019a; Ježek et al. 2020).

###### Note.

First record for Azerbaijan.

##### Pericoma (Pericoma) pseudoexquisita

Taxon classificationAnimaliaDipteraPsychodidae

Tonnoir, 1940

8B576D47-BD3D-5DF5-A4AA-4F790DA35ED4

###### Material examined.

**Georgia**: G 11, 3.5.2019, 1♂, slide Inv. No. 25674, leg. JO; G 16, 2.5.2019, 1♂, slide Inv. No. 25680, leg. JO.

###### Distribution.

Species known from the whole of Europe: Austria, Belgium, Bulgaria, Czech Republic, France, Germany, Greece, Great Britain, Hungary, Ireland, Italy, Slovakia, Slovenia, Spain, and Switzerland (Ježek et al. 2018b; Wagner 2018).

###### Note.

First record for Georgia and Transcaucasia.

##### Pneumia canescens

Taxon classificationAnimaliaDipteraPsychodidae

(Meigen, 1804)

DB7C6AF7-08A0-5BAF-8E90-87C62061F86C

###### Material examined.

**Azerbaijan**: A 03, 1.10.2019, 1♂, slide Inv. No. 25588, leg. PM. **Georgia**: G 12, 9.7.2019, 1♂, slide Inv. No. 25612, leg. PM, GV.

###### Distribution.

This is a common European and western Siberian species. In Europe it is known from Austria, Belgium, Great Britain, Czech Republic, Denmark, European Turkey, France, Germany, Greece, Hungary, Slovakia, Sweden, and the Netherlands. In Asia, it is known from Armenia, Turkey, Kyrgyzstan, Afghanistan, and China; it occurs from lowlands to mountains, usually associated with mosses in running water habitats; its larvae are rheobiotic (Ježek 2006; Omelková and Ježek 2012; Ježek et al. 2013, 2018b).

###### Note.

First record for Azerbaijan and Georgia.

##### Pneumia gracilis gracilis

Taxon classificationAnimaliaDipteraPsychodidae

(Eaton, 1893)

0A5DC85E-BD94-5355-80BB-99E617E6182C

###### Material examined.

**Georgia**: G 02, 24.9.2019, 1♂, slide Inv. No. 25585, leg. TK, PM.

###### Distribution.

The nominal subspecies was recorded from several European countries and is also known from Abkhazia in the Transcaucasia; it lives in forest slope spring areas, brooks, and marshes (Ježek 2002, 2004; Ježek et al. 2012, 2017; Ježek and Omelková 2012; Omelková and Ježek 2012).

###### Note.

First records for Georgia outside of Abkhazia.

##### Pneumia nubila

Taxon classificationAnimaliaDipteraPsychodidae

(Meigen, 1818)

D4D81DFF-CF9F-5D43-8F82-25FB89D88D49

###### Material examined.

**Azerbaijan**: A 02, 9.5.2019, 1♂, slide Inv. No. 25659, leg. JO; A 07, 30.9.2019, 1♂, slide Inv. No. 25590, leg. PM; A 08, 30.9.2019, 1♂, slide Inv. No. 25602, leg. PM; A 13, 7.5.2019, 1♂, slide Inv. No. 25741, leg. JO; A 21, 8.5.2019, 1♂, slide Inv. No. 25750, leg. JO; A 23, 6.5.2019, 1♂, slide Inv. No. 25684, leg. JO. **Georgia**: G 09, 2.5.2019, 1♂, slide Inv. No. 25641, leg. JO; G 25, 29.4.2019, 1♂, slide Inv. No. 25712, leg. JO.

###### Distribution.

This is a very common species which is recorded from throughout Europe, Armenia, and the Canary Islands. In Europe, it is known from Austria, Belgium, Bosnia and Herzegovina, Bulgaria, Croatia, Czech Republic, Denmark, Finland, France, Georgia, Germany, Great Britain, Greece, Hungary, Ireland, Italy, Luxembourg, Macedonia, Montenegro, Poland, Romania, Sardinia, Serbia, Slovakia, Slovenia, Spain, Switzerland, the Netherlands, and Ukraine; abundant especially in shaded habitats with decaying organic matter, e.g., ponds, brooks, spring areas, water reservoirs, and swamps (Wagner 1981; Ježek and Goutner 1995; Krek 1999; Ježek 2002; Omelková and Ježek 2012; Kvifte et al. 2013; Ježek et al. 2017, 2018a).

###### Note.

First record for Azerbaijan.

##### Pneumia palustris

Taxon classificationAnimaliaDipteraPsychodidae

(Meigen, 1804)

6A9D0B55-3FB6-5196-9DCF-FE6C2B0371FB

###### Material examined.

**Georgia**: G 24, 29.4.2019, 1♂, slide Inv. No. 25677, leg. JO; G 48, 28.4.2019, 1♂, slide Inv. No. 25646, leg. JO.

###### Distribution.

European species, also registered from Turkey, Georgia, and the Canary Islands (Kroča and Ježek 2019; Oboňa et al. 2019).

##### Pneumia pilularia

Taxon classificationAnimaliaDipteraPsychodidae

(Tonnoir, 1940)

F3427769-B8AB-511D-AA57-0B4121DB1E04

###### Material examined.

**Azerbaijan**: A 03, 2.10.2019, 1♂, slide Inv. No. 25587, leg. PM; A 04, 1.10.2019, 1♂, slide Inv. No. 25593, leg. PM; A 09, 30.9.2019, 1♂, slide Inv. No. 25582, leg. PM; A 10, 6.5.2019, 2♂♂, slide Inv. No. 25647 and 25675, leg. JO; A 12, 8.5.2019, 1♂, slide Inv. No. 25698, leg. JO; A 20, 5.5.2019, 1♂, slide Inv. No. 25721, leg. JO. **Georgia**: G 16, 2.5.2019, 1♂, slide Inv. No. 25679, leg. JO; G 29, 30.9.2019, 1♂, slide Inv. No. 25600, leg. GV; G 36, 27.4.2019, 1♂, slide Inv. No. 25667, leg. JO; G 39, 9.7.2019, 1♂, slide Inv. No. 25705, leg. PM; G 42, 4.7.2019, 1♂, slide Inv. No. 25620, leg. PM; G 44, 10.7.2019, 1♂, slide Inv. No. 25628, leg. TK, PM, DM; G 45, 10.7.2019, 1♂, slide Inv. No. 25607, leg. TK, PM, DM. JO; G 46, 1.10.2019, 1♂, slide Inv. No. 25608, leg. GV; G 49, 28.4.2019, 1♂, slide Inv. No. 25669, leg. JO; G 50, 28.4.2019, 1♂, slide Inv. No. 25656, leg. JO.

###### Distribution.

European species. Known also from Tajikistan, Azerbaijan, and Georgia (Kroča and Ježek 2019; Oboňa et al. 2019a).

##### Pneumia trivialis

Taxon classificationAnimaliaDipteraPsychodidae

(Eaton, 1893)

8F7362A2-E887-529D-AA04-5FDA8FEE0AF1

###### Material examined.

**Azerbaijan**: A 21, 8.5.2019, 1♂, slide Inv. No. 25725, leg. JO. **Georgia**: G 24, 29.4.2019, 1♂, slide Inv. No. 25744, leg. JO.

###### Distribution.

A very common European species. Known from Austria, Belgium, Bosnia and Herzegovina, Croatia, Czech Republic, Denmark, Finland, France, Germany, Great Britain, Hungary, Ireland, the Netherlands, Norway, Poland, Serbia, Slovakia, Slovenia, Spain, Sweden, Switzerland, and Ukraine, in both shaded and unshaded habitats with decaying organic matter (ponds, brooks, spring areas, swamps, and water reservoirs) where larvae develop (Krek 1999; Ježek 2002; Kvifte et al. 2011, 2013; Omelková and Ježek 2012; Ježek et al. 2017).

###### Note.

First record for Azerbaijan, Georgia, and Transcaucasia.

##### Psycha grisescens

Taxon classificationAnimaliaDipteraPsychodidae

(Tonnoir, 1922)

68F71813-E169-5627-835F-D0888D9DC3CF

###### Material examined.

**Azerbaijan**: A 23, 6.5.2019, 1♂, slide Inv. No. 25691, leg. JO.

###### Distribution.

Species known throughout Europe, including northern areas (British Isles, Scandinavian bioregion) and Central European countries, penetrating eastwards as far as Turkey (Anatolia) and Azerbaijan; southern frontier of distribution is limited by North Africa (Kroča and Ježek 2019; Oboňa et al. 2019a).

##### Psychoda phalaenoides

Taxon classificationAnimaliaDipteraPsychodidae

(Linné, 1758)

BB20353A-CC95-5088-91E3-643F75B8A914

###### Material examined.

**Azerbaijan**: A 02, 9.5.2019, 1♂, slide Inv. No. 25666, leg. JO.

###### Distribution.

Widespread Holarctic polyvoltine species occurring from lowlands to mountains; known also from Georgia (e.g., Ježek 1990b; Oboňa and Ježek 2014; Oboňa et al. 2019a).

###### Note.

First record for Azerbaijan.

##### Psychodocha cinerea

Taxon classificationAnimaliaDipteraPsychodidae

(Banks, 1894)

33C1F923-768D-5F1B-9124-D53D6F5F8279

###### Material examined.

**Azerbaijan**: A 20, 5.5.2019, 1♂, slide Inv. No. 25722, leg. JO; A 23, 6.5.2019, 2♀♀, slide Inv. No. 25686 and 25694, leg. JO. **Georgia**: G 10, 4.5.2019, 1♀, slide Inv. No. 25711, leg. JO.

###### Distribution.

A very common cosmopolitan species ranging from lowlands to mountains. The larvae are saprobiotic, occasionally associated with fungi; the adults are often found in unclean bathrooms. Known from Austria, Azores, Belgium, Bosnia and Herzegovina, Bulgaria, the Canary Islands, Cyprus, Czech Republic, Denmark, Finland, France, Germany, Great Britain, Greece, Hungary, Ireland, Italy (incl. Sardinia), Madeira, the Netherlands, Norway, Poland, Romania, Russia, Serbia, Slovakia, Slovenia, Spain, Sweden, Switzerland, Ukraine, Turkey, Abkhazia, Afghanistan, Africa mer., Algeria, Argentina, Australia, Azores, Brazil, Canada, Chile, Iran, Israel, Juan Fernandéz Islands, New Zealand, Puerto Rico Islands, Tunisia, USA (Krek 1985; Wagner 1990, 2018; Ježek and Goutner 1995; Ježek and Yağci 2005; Kvifte et al. 2011; Salmela et al. 2014; Ježek et al. 2017).

###### Note.

First record for Azerbaijan. First record for Georgia, from a territory other than Abkhazia.

##### Psychodocha gemina

Taxon classificationAnimaliaDipteraPsychodidae

(Eaton, 1904)

3828E873-A56E-5E71-9F31-88E14080E682

###### Material examined.

**Azerbaijan**: A 05, 1.10.2019, 1♀, slide Inv. No. 25579, leg. PM; A 23, 6.5.2019, 1♀, slide Inv. No. 25685, leg. JO. **Georgia**: G 09, 2.5.2019, 1♀, slide Inv. No. 25733, leg. JO; G 19, 30.4.2019, 1♀, slide Inv. No. 25651, leg. JO; G 24, 29.4.2019, 1♂, 1♀, slide Inv. No. 25735 and 25748, leg. JO.

###### Distribution.

A common European species, it occurs commonly from lowlands to mountains. The larvae are saprobiotic and often develop in nests of water birds. Recorded from Austria, Belgium, Bosnia and Herzegovina, Croatia, Czech Republic, Great Britain, Denmark, France, Finland, Germany, Greece, Hungary, Ireland, Norway, Romania, Serbia, Slovakia, Slovenia, Spain, Switzerland, the Netherlands, Ukraine, and Georgia: Abkhazia (Ježek and Goutner 1995; Krek 1999; Ježek 2002; Kvifte et al. 2011, 2013; Ježek and Omelková 2012; Salmela et al. 2014; Ježek et al. 2017; Oboňa et al. 2019a).

###### Note.

First record for Azerbaijan.

##### Psychomora mycophila

Taxon classificationAnimaliaDipteraPsychodidae

(Vaillant, 1988)

33904ADD-0DEA-5925-8977-8DC5099E364A

###### Material examined.

**Azerbaijan**: A 23, 6.5.2019, 1♂, slide Inv. No. 25690, leg. JO; **Georgia**: G 05, 25.9.2019, 1♂, slide Inv. No. 25589, leg. PM.

###### Distribution.

A rare species associated with fungi, occurs from lowlands to mountains, so far known only from the Czech Republic, France, Slovakia, Slovenia, Switzerland, and Ukraine (Ježek and Omelková 2012; Ježek et al. 2017).

###### Note.

First record for Azerbaijan, Georgia, and Transcaucasia.

##### Psychomora trinodulosa

Taxon classificationAnimaliaDipteraPsychodidae

(Tonnoir, 1922)

7CEA480C-520B-5F43-81B1-E99A4354A96D

###### Material examined.

**Azerbaijan**: A 19, 7.5.2019, 1♂, slide Inv. No. 25724, leg. JO. **Georgia**: G 22, 15.7.2019, 1♂, slide Inv. No. 25599, leg. TK, DM, GV.

###### Distribution.

A very common Holarctic species. Known from Austria, Belgium, Bulgaria, Croatia, Czech Republic, Denmark, Finland, France, Germany, Great Britain, the Greek mainland, Hungary, Ireland, Italy, the Netherlands, Norway, Poland, Romania, Russia, Sardinia, Slovakia, Slovenia, the Spanish mainland, Sweden, Ukraine, Georgia, Algeria, and USA (Ježek 1990c; Wagner 2013; Salmela et al. 2014; Ježek et al. 2017; Oboňa et al. 2019a).

###### Note.

First record for Azerbaijan. It is a vector of larval stages of *Rhabditis* nematodes and Gamasidae mites (Ježek and Omelková 2012).

##### Saraiella ressli

Taxon classificationAnimaliaDipteraPsychodidae

Wagner, 1981

EC8AE2A0-B482-5390-BE5F-EA33C55F519B

S. ressli montana Ježek, 1990, syn. nov.

###### Material examined.

**Azerbaijan**: A 02, 9.5.2019, 1♂, slide Inv. No. 25660, leg. JO; A 04, 1.10.2019, 1♂, slide Inv. No. 25595, leg. PM; A 05, 1.10.2019, 1♂, slide Inv. No. 25576, leg. PM; A 06, 1.10.2019, slide Inv. No. 25573, 1♂, leg. JJ, PM; A 10, 6.5.2019, 2♂♂, slide Inv. No. 25676 and 25648, leg. JO; A 14, 7.5.2019, 1♂, slide Inv. No. 25737, leg. JO; A 16, 10.5.2019, 1♂, slide Inv. No. 25643, leg. JO; A 17, 10.5.2019, 1♂, slide Inv. No. 25700, leg. JO; A 18, 10.5.2019, 1♂, slide Inv. No. 25645, leg. JO; A 23, 6.5.2019, 1♂, slide Inv. No. 25692, leg. JO; A 24, 9.5.2019, 1♂, slide Inv. No. 25703, leg. JO. **Georgia**: G 03, 27.9.2019, 1♂, slide Inv. No. 25606, leg. PM; G 04, 27.9.2019, 1♂, slide Inv. No. 25592, leg. PM; G 09, 2.5.2019, 2♂♂, slides Inv. No. 25640, 25726, leg. JO; G 12, 9.7.2019, 1♂, slide Inv. No. 25614, leg. PM, GV; G 14, 2.5.2019, 1♂, slide Inv. No. 25658, leg. JO; G 15, 2.5.2019, 2♂♂, slides Inv. No. 25673, 25702 leg. JO; G 16, 28.9.2019, 1♂, slide Inv. No. 25586, leg. GV; G 31, 27.4.2019, 1♂, slide Inv. No. 25697, leg. JO; G 37, 2.10.2019, 1♂, slide Inv. No. 25701, leg. PM; G 38, 27.4.2019, 1♂, slide Inv. No. 25704, leg. JO; G 40, 13.7.2019, 1♂, slide Inv. No. 25632, leg.TK, PM, DM, GV; G 47; 9.7.2019, 1♂, slide Inv. No. 25636, leg. TK, PM, DM, GV; G 49, 28.4.2019, 1♂, slide Inv. No. 25668, leg. JO; G 50, 28.4.2019, 1♂, slide Inv. No. 25655, leg. JO; G 51, 28.4.2019, 1♂, slide Inv. No. 25699, leg. JO; G 52, 11.7.2019, 1♂, slide Inv. No. 25618, leg. TK, PM, DM, GV; G 53, 9.7.2019, 1♂, slide Inv. No. 25623, leg. TK, PM, DM, GV; G 54, 27.4.2019, 1♂, slide Inv. No. 25672, leg. JO; G 55, 29.9.2019, 1♂, slide Inv. No. 25580, leg. PM.

###### Distribution.

Species known from Iran, Armenia, Azerbaijan, and Russia (Wagner and Joost 1983; Ježek et al. 2018). First record for Georgia.

###### Note.

*Saraiella ressli* Wagner, 1981, was described only on the basis of a holotype from the environment of the Caspian Sea (northern Iran, Veyshar nr. Nowshahr env. Chalus – Mazandaran, 1400 m a.s.l.). *Saraiella ressli montana* Ježek, 1990, was described from S. E. Iran, Kerman province, Kuh-e-Lalehzar, 3850–4374 m a.s.l. with top 4374 m (holotype and two paratypes), and additionally two paratypes from E. Iran, Kerman province, Deh Bakri, Kuh-e Jebal Barez (Jebal Barez Mts). The numerous materials from Caucasus (males) cited above proved a large variability of specimens (last three flagellomeres almost globular or long-oval, terminal flagellomere with small apiculus or without a protuberance, coxopodites basally with or without several bristles, gonostylus with a small subapical tooth terminally or not; all characters are mixed on the same localities). The subspecific rank is therefore groundless and *S. ressli montana* Ježek, 1990 is thus sunk as a synonym herein.

##### Saraiella rotunda

Taxon classificationAnimaliaDipteraPsychodidae

(Krek, 1970)

770EAF0E-3A29-5B60-8519-0C26F5AA7306

###### Material examined.

**Azerbaijan**: A 04, 1.10.2019, 1♂, slide Inv. No. 25594, leg. PM, A 06, 1.10.2019, 1♂, slide Inv. No. 25572, leg. PM. **Georgia**: G 07, 17.7.2019, 1♂, slide Inv. No. 25591, leg. GV.

###### Distribution.

European species, known from Bosnia and Herzegovina, Czech Republic, Italy, Poland, Serbia, and Slovakia (Ježek 2006; Oboňa et al. 2019b). Occurs in spring areas and swamps, forest edge in Slovakia (High Tatras Mts.) (Ježek 2006).

###### Note.

First record for Azerbaijan, Georgia, and Transcaucasia.

##### Seoda svanetica

Taxon classificationAnimaliaDipteraPsychodidae

(Ježek, 1988)

785DAD40-2B24-5A41-A534-F61C157772D5

###### Material examined.

**Georgia**: G 09, 2.5.2019, 1♂, slide Inv. No. 25729, leg. JO.

###### Distribution.

Species known only from the original paper from Abkhazia, where only one specimen was collected on the bank of a stream in 2000 m a.s.l. (Ježek 1988 as *Telmatoscopus*).

###### Note.

First record since the original description. First record for Georgia, a territory other than Abkhazia. An extremely rare species known from only two sites in Greater Caucasus.

##### Thornburghiella veve

Taxon classificationAnimaliaDipteraPsychodidae

Oboňa & Ježek, 2017

7D47C182-E67C-5C59-A1A1-1160B31E8145

###### Material examined.

**Azerbaijan**: A 05, 1.10.2019, 1♂, slide Inv. No. 25575, leg. PM.

###### Distribution.

Species known only from the original paper from Armenia (Oboňa et al. 2017).

###### Note.

First record since the original description, first record for Azerbaijan. An extremely rare species known from only two sites in the Lesser Caucasus at elevations higher than 1000 m.

##### Threticus balkaneoalpinus

Taxon classificationAnimaliaDipteraPsychodidae

Krek, 1972

0782973D-0244-5717-A669-71A51EF760AA

###### Material examined.

**Georgia**: G 09, 2.5.2019, 1♂, slide Inv. No. 25728, leg. JO; G 30, 13.7.2019, 1♂, slide Inv. No. 25611, leg. TK, PM, DM, GV.

###### Distribution.

Species known from Austria, Bosnia, Czech Republic, France, Germany, Kosovo, Poland, Slovakia, Switzerland, and the United Kingdom as well as Abkhazia (Krek 1990; Ježek 1995; Withers 1997; Ježek and Omelková 2007; Oboňa and Ježek 2014; Oboňa et al. 2019b).

###### Note.

First record for Georgia, territory other than Abkhazia.

##### Threticus petrosus

Taxon classificationAnimaliaDipteraPsychodidae

Ježek, 1997

0BDD7D38-2FED-521A-BDFC-2A498D4DC2F3

###### Material examined.

**Georgia**: G 06, 24.9.2019, 1♂, slide Inv. No. 25598, leg. GV.

###### Distribution.

Known only from the original paper from Abkhazia (Ježek 1997; Bzybskij khrebet, pastoral community Kot-Kot nr. peak Khimsa (3033 m a.s.l.) – holotype + 9 paratypes (males)).

###### Note.

First record since the original description. First record for Georgia, from a territory other than Abkhazia. An extremely rare species known from only two sites at a distance ca. 20–30 km from the Black Sea coast at elevations of ca. 2000 and 2600 m.

##### Tinearia alternata

Taxon classificationAnimaliaDipteraPsychodidae

(Say, 1824)

38FFC610-53BF-50CE-AC93-10E49FFAD9C4

###### Material examined.

**Georgia**: G 03, 27.9.2019, 1♀, slide Inv. No. 25603, leg. PM; G 08, 1.5.2019, 1♀, slide Inv. No. 25683, leg. JO; g 56, 26.4.2019, 1♀, slide Inv. No. 25682, leg. JO.

###### Distribution.

Cosmopolitan and euryvalent species (Kroča and Ježek 2019).

##### Tonnoiriella arcuata

Taxon classificationAnimaliaDipteraPsychodidae

Ježek, 1997

EDBEBC77-50D2-5348-8DB0-EF614CCF4142

###### Material examined.

**Azerbaijan**: A 17, 10.5.2019, 2♂♂, slide Inv. No. 25718 and 25719, leg. JO. **Georgia**: G 09, 2.5.2019, 1♂, slide Inv. No. 25730, leg. JO; G 40, 13.7.2019, 1♂, slide Inv. No. 25635, leg. TK, PM, DM, GV; G 42, 4.7.2019, 1♂, slide Inv. No. 25622, leg. PM; G 47, 9.7.2019, 1♂, slide Inv. No. 25637, leg. TK, PM, DM, GV.

###### Distribution.

Species known only from the original paper from Abkhazia (Ježek 1997; Pskhu), holotype + 6 paratypes (males).

###### Note.

First record since the original description, first record for Azerbaijan. Occurs at a wide range of elevations (810–3050 m a.s.l.) at small forest or bushy streams, springs, and side brooks of the Greater Caucasus.

##### Ulomyia cognata

Taxon classificationAnimaliaDipteraPsychodidae

(Eaton, 1893)

18282418-0BAA-5FB0-A92A-66697C738834

###### Material examined.

**Georgia**: G 27, 17.7.2019, 1♂, slide Inv. No. 25570, leg. GV; G 33, 5.7.2019, 1♂, slide Inv. No. 25629, leg. PM, GV; G 40, 13.7.2019, 1♂, slide Inv. No. 25631, leg. TK, PM, DM, GV; G 42, 4.7.2019, 2♂♂, slide Inv. No. 25619 and 25621, leg. PM; G 47, 9.7.2019, 1♂, slide Inv. No. 25639, leg. TK, PM, DM, GV.

###### Distribution.

This is a common European species known from Austria, Czech Republic, Finland, France, Germany, Great Britain, Italy, Lithuania, Poland, Slovakia, Slovenia, Ukraine, and Armenia (Ježek and Omelková 2012; Salmela et al. 2014; Ježek et al. 2017, 2018a).

###### Note.

First record for Georgia. In Europe, a very common species from lowlands to mountains, but in Georgia it has been found only in mountain localities at an elevation of 2050 to 3050 m a.s.l.

##### Yomormia afonensis

Taxon classificationAnimaliaDipteraPsychodidae

Ježek, 1987

0FE423EB-3D6D-5D1A-A3B5-ACB97D945453

###### Material examined.

**Azerbaijan**: A 15, 8.5.2019, 1♂, slide Inv. No. 25706, leg. JO. **Georgia**: G 19, 30.4.2019, 1♂, slide Inv. No. 25650, leg. JO.

###### Distribution.

Species known only from the original paper from Abkhazia (Ježek 1987; only on the basis of a holotype (male) from Kolchidian lowland (Novyj Afon) at the Black Sea coast).

###### Note.

First records since the original description, first record for Azerbaijan and Georgia, from a territory other than Abkhazia. An extremely rare species known from only three considerably remote localities in lowland and foothills of the Greater Caucasus (recent records at 655 and 845 m a.s.l.).

## Discussion

Although faunistic research of the family Psychodidae has been the subject of a number of authors (see the Introduction for references) and a large number of sites were examined in our recent studies (Ježek et al. 2014, 2018; Oboňa et al. 2017, 2019a), each of our subsequent expeditions has highlighted species new for science and a number of new records for the Caucasus (or Transcaucasia) or for countries where we carried out the sampling. This study is no exception, and in addition to species new to science (Ježek et al. 2021), we provide faunistic data on 46 species of the family Psychodidae in this publication, up to 12 of which are first records for Transcaucasia, 22 for Azerbaijan, and 17 for Georgia. A total of 80 sampling sites was sampled during the 2019 campaign (24 in Azerbaijan and 56 in Georgia). Therefore, this is another significant shift in the knowledge of the faunistics of this family, but also in regard to the biogeographical and ecological aspects of several recorded species.

One of our most interesting results is that we confirmed several species that were described from the Abkhazia region, and no one has been able to confirm them anywhere else until this study was carried out, despite several sampling campaigns. These are species that are extremely rare or relatively common in Abkhazia. Extremely rare was *Seoda svanetica* (Ježek 1988), of which we recorded only other one male. Other extremely rare species known only from Abkhazia until now are *Threticus petrosus* Ježek, 1997, now known from only two sites, and *Yomormia afonensis* Ježek, 1987, which has been found at only three localities. The species *Tonnoiriella arcuata* Ježek, 1997, was also very rare in Abkhazia, but we found this species at five other sites located at a large range of elevations in the Greater Caucasus. Other species were not considered rare in Abkhazia, for example *Sycorax caucasica* Ježek, 1990, *Mormia ckvitariorum* Ježek, 1987, and *Seoda svanetica* (Ježek, 1988), but we found them at only one site and only one (*S. caucasica*, *S. svanetica*) to three (*M. ckvitariorum*) males. This could be caused by the great rarity of these species in other parts of Transcaucasia or in the change that the fauna has undergone under the influence of various factors during the 30 years that divide the records from Abkhazia and our recent ones. However, these assumptions are based on the results obtained using the collection methods described above. Therefore, in order to answer this question definitively, it is necessary to carry out research using more appropriate collection methods and corresponding statistical analyses.

Based on our new data, knowledge of some aspects of the ecology and biogeography of selected (especially rare) species has also expanded. Our records point out that several rare species show different patterns related to elevation: Clytocerus (Boreoclytocerus) grusinicus and *Tonnoiriella arcuata* occur at large range of elevations, *Mormia ckvitariorum* and *Yomormia afonensis* prefer low elevations in lowlands and low foothills, *Thornburghiella veve* elevations higher than 1000 m, and *Threticus petrosus* sites at elevations higher than 2000 m relatively close to the Black Sea coast. Several species considered as European (or rare European) occur at numerous localities in the Transcaucasia and should be reconsidered as European and Transcaucasian species (*Promormia silesiensis*, *Panimerus denticulatus*, Pericoma (Pachypericoma) nielseni).

**Figure 8.**
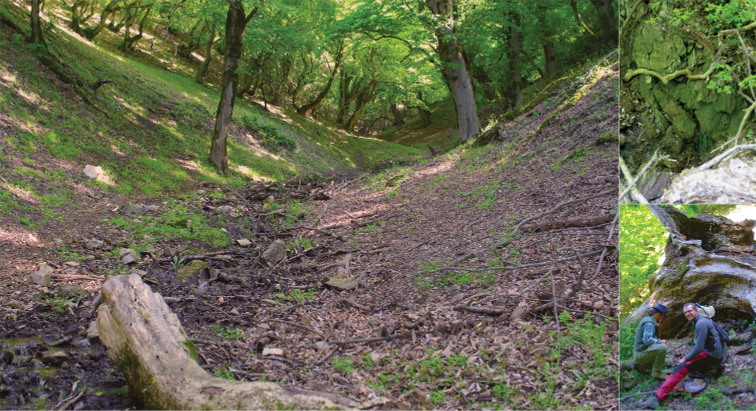
The collecting site with the highest species richness in Azerbaijan (A 23), a karst brook in deciduous forest in the Şəki district, Şəki, Quirxbulaq, with seven species collected; general view (left) and different habitats (top right and bottom right which also include colleagues Libor Dvořák (left) and Ľuboš Hrivniak (right) collecting insects during a joint sampling campaign); photograph P. Manko.

**Figure 9.**
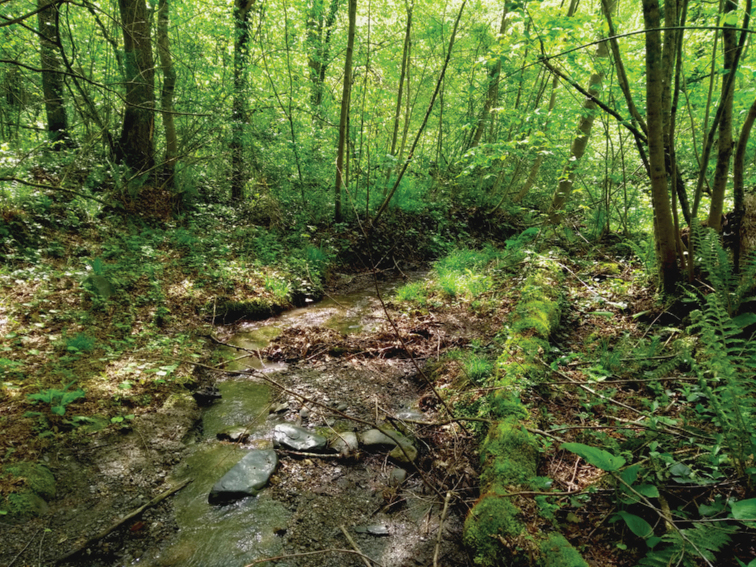
The collecting site with the highest species richness in Georgia (G 09), Batsara River and its side brook, Kakheti region, Batsara Nature Reserve, with nine species collected; photograph P. Manko.

Although we usually found only one to three species in most sites located in cultivated and managed areas, mosaic croplands and mostly open areas with dominant herbaceous cover, the species number was quite high at other sampling sites. The highest species richness was recorded at sites A 23 (Fig. 8) and G 09 (Fig. 9), which are located at relatively low elevations along watercourses flowing through preserved deciduous forests (G 09 in Nature Reserve) and characterized by a high diversity of habitats. Also, most of the other sampling sites with high diversity are located in forests (e.g., A 02, G 09, G 40) or rich lush shrub vegetation (e.g., A 05). It is also interesting that many of the first records and records of rare species were obtained from the above-mentioned (close to) natural sites with highest species richness (A 02 with seven spp, and A 23 and G 09 with nine spp each).

## Conclusions

Out of the total number of 46 species/subspecies (Sycoracinae 1 sp., Trichomyiinae 1 sp. Psychodinae 44 sp.) and 182 slides, 12 species are recorded for the first time for Transcaucasia (namely *Copropsychoda brevicornis*, *Feuerborniella obscura*, *Logima sigma*, *L. zetterstedti*, *Panimerus denticulatus*, Pericoma (Pachypericoma) nielseni, P. (Pericoma) pseudoexquisita, *Pneumia trivialis*, *Promormia silesiensis*, *Psychomora mycophila*, *Saraiella rotunda*, *Trichomyia urbica*), 22 species for Azerbaijan (namely Clytocerus (Boreoclytocerus) grusinicus, *Logima sigma*, *Mormia ckvitariorum*, *Panimerus denticulatus*, Pericoma (Pachypericoma) nielseni, *Pericoma* (*Pericoma*.) *kariana*, P. (P.) motasi motasi, *Pneumia canescens*, *P. nubila*, *P. trivialis*, *Promormia silesiensis*, *Psychoda phalaenoides*, *Psychodocha cinerea*, *P. gemina*, *Psychomora mycophila*, *P. trinodulosa*, *Saraiella ressli*, *S. rotunda*, *Thornburghiella veve*, *Tonnoiriella arcuata*, *Trichomyia urbica*, *Yomormia afonensis*), 17 species for Georgia (*Copropsychoda brevicornis*, *Feuerborniella obscura*, *Logima albipennis*, *L. zetterstedti*, *Panimerus denticulatus*, *Parabazarella joosti lalehzarica*, Paramormia (Duckhousiella) ustulata, Pericoma (Pachypericoma) nielseni, Pericoma (Pericoma) bosniaca, P. (P.) exquisita, P. (P.) pseudoexquisita, *Pneumia canescens*, *P. trivialis*, *Psychomora mycophila*, *Saraiella ressli*, *S. rotunda*, *Ulomyia cognata*), and nine species for the first time since their original descriptions (Clytocerus (Boreoclytocerus) grusinicus, *Mormia ckvitariorum*, *Parabazarella joosti lalehzarica*, Pericoma (P.) kariana, *Sycorax caucasica*, *Thornburghiella veve*, *Threticus petrosus*, *Tonnoiriella arcuata*, *Yomormia afonensis*). The knowledge of some aspects of ecology and biogeography of selected (especially rare) species has been expanded and a clear pattern was found in species richness, rare species, and new records in relation to land use, habitat diversity and preservation of the environment surrounding the sampling sites.

## Supplementary Material

XML Treatment for Sycorax caucasica

XML Treatment for Trichomyia urbica

XML Treatment for Clytocerus (Boreoclytocerus) grusinicus

XML Treatment for Copropsychoda brevicornis

XML Treatment for Feuerborniella obscura

XML Treatment for Chodopsycha lobata

XML Treatment for Logima albipennis

XML Treatment for Logima satchelli

XML Treatment for Logima sigma

XML Treatment for Logima zetterstedti

XML Treatment for Mormia ckvitariorum

XML Treatment for Promormia silesiensis

XML Treatment for Panimerus denticulatus

XML Treatment for Paramormia (Duckhousiella) ustulata

XML Treatment for Parabazarella joosti lalehzarica

XML Treatment for Pericoma (Pachypericoma) blandula

XML Treatment for Pericoma (Pachypericoma) fallax

XML Treatment for Pericoma (Pachypericoma) nielseni

XML Treatment for Pericoma (Pericoma) bosniaca

XML Treatment for Pericoma (Pericoma) exquisita

XML Treatment for Pericoma (Pericoma) kariana

XML Treatment for Pericoma (Pericoma) motasimotasi

XML Treatment for Pericoma (Pericoma) pseudoexquisita

XML Treatment for Pneumia canescens

XML Treatment for Pneumia gracilis gracilis

XML Treatment for Pneumia nubila

XML Treatment for Pneumia palustris

XML Treatment for Pneumia pilularia

XML Treatment for Pneumia trivialis

XML Treatment for Psycha grisescens

XML Treatment for Psychoda phalaenoides

XML Treatment for Psychodocha cinerea

XML Treatment for Psychodocha gemina

XML Treatment for Psychomora mycophila

XML Treatment for Psychomora trinodulosa

XML Treatment for Saraiella ressli

XML Treatment for Saraiella rotunda

XML Treatment for Seoda svanetica

XML Treatment for Thornburghiella veve

XML Treatment for Threticus balkaneoalpinus

XML Treatment for Threticus petrosus

XML Treatment for Tinearia alternata

XML Treatment for Tonnoiriella arcuata

XML Treatment for Ulomyia cognata

XML Treatment for Yomormia afonensis

## Supplementary materials

Supplementary material 1 Table S1. List of localities with altitudes and coordinates Data type occurrence https://binary.pensoft.net/file/567182This dataset is made available under the Open Database License (http://opendatacommons.org/licenses/odbl/1.0/). The Open Database License (ODbL) is a license agreement intended to allow users to freely share, modify, and use this Dataset while maintaining this same freedom for others, provided that the original source and author(s) are credited.Jan Ježek, Peter Manko, Jozef Oboňa
