# Supplementary material for: Psychodidae (Diptera) of Azerbaijan and Georgia – faunistics with biodiversity notes
Source: Zookeys. 2021 Jun 15;1049:15–42. doi: 10.3897/zookeys.1049.66063 (PMC8298371; doi:10.3897/zookeys.1049.66063)
Supplement: Supplementary material 1 — Table S1. List of localities with altitudes and coordinates [file zookeys-1049-015-s001.docx]

Supplementary Table 1

**List of localities with altitudes and coordinates**

**Azerbaijan**

A 01, Balakǝn district, Balakǝn, Mahamalar, open brook and marsh, 465 m a.s.l., 41°44'13.1"N 46°26'13.9"E

A 02, Balakǝn district, forest brook, 320 m a.s.l., 41°40'35.9"N 46°29'40.7"E

A 03, Dașkəsən district, Xoșbulaq, open brook S of the village, 1710 m a.s.l., 40°26'01.2"N 46°02'46.6"E

A 04, Gədəbəy district, Gədəbəy, big stream S of the village, 1510 m a.s.l., 40°27'13.1"N 45°43'02.7"E

A 05, Gədəbəy district, Gədəbəy, bushy brook and seep S of the village, 1500 m a.s.l., 40°27'31.1"N 45°43'06.8"E

A 06, Gədəbəy district, Gədəbəy, open brook and seep S of the village, 1480 m a.s.l., 40°27'36.1"N 45°43'08.6"E

A 07, Göygöl district, Göygöl N.P., forest brook below Maralgöl Lake, 1875 m a.s.l., 40°22'51.3"N 46°18'30.4"E

A 08, Göygöl district, Göygöl N.P., Göygöl Lake, 1570 m a.s.l., 40°24'53.2"N 46°19'39.6"E

A 09, Göygöl district, Toğanalı, Kürǝkçay Stream, 1250 m a.s.l., 40°25'07.0"N 46°18'12.2"E

A 10, Oğuz district, Baş Daşağıl waterfall and brook in deciduous forest, 1325 m a.s.l., 41°10'55.0"N 47°23'43.0"E

A 11, Oğuz district, Sincan, open river, 370 m a.s.l., 40°56'02.8"N 47°34'55.0"E

A 12, Qax district, Ilisu, Ilisu Waterfall and the stream beneath, 1478 m a.s.l., 41°27'30.3"N 47°04'13.0"E

A 13, Qax district, Lǝkit, large stream and its littoral, 530 m a.s.l., 41°29'16.9"N 46°51'20.9"E

A 14, Qax district, Lǝkit, Mamırlı Waterfall and springs, 600 m a.s.l., 41°29'34.0"N 46°51'32.1"E

A 15, Qax district, Qum, forest brooks above the village, 845 m a.s.l., 41°28'12.9"N 46°55'58.6"E

A 16, Qǝbǝlǝ district, Durca, Dəmiraparançay Stream and forest brooks, 1595 m a.s.l., 41°03'13.6"N 47°53'12.1"E

A 17, Qǝbǝlǝ district, Durca, forest edge and brook, 1310 m a.s.l., 41°02'15.9"N 47°53'11.7"E

A 18, Qǝbǝlǝ district, Laza, springs and brooks on the opposite slope, 1435 m a.s.l., 41°02'23.9"N 47°55'53.5"E

A 19, Şəki district, Cumakənd, Əyriçay River, marsh and open woods, 200 m a.s.l., 41°14'13.4"N 46°54'36.1"E

A 20, Şəki district, Kiş, forest beneath Gələrsən-Görәrsәn ruin, 1190 m a.s.l., 41°15'44.6"N 47°13'37.0"E

A 21, Şəki district, Kiş, forest brook above the village, 1050 m a.s.l., 41°15'36.3"N 47°11'08.6"E

A 22, Şəki district, Kiş, hotel garden, 905 m a.s.l., 41°14'39.9"N 47°11'22.6"E

A 23, Şəki district, Şəki, Quırxbulaq, karst brook in deciduous forest, 595 m a.s.l., 41°08'47.2"N 47°15'31.9"E (Fig. 8)

A 24, Zaqatala district, Car, Talaçay Stream above the village, 950 m a.s.l., 41°40'40.7"N 46°41'57.9"E

**Georgia**

G 01, Adjara, Khichauri, Chvanis Tskali River below the bridge, 350 m a.s.l., 41°38'42.9"N 42°07'59.4"E

G 02, Adjara, Kintrishi River and its sidespring at Varjanauli Bridge, 215 m a.s.l., 41°47'19.4"N 41°57'37.9"E

G 03, Adjara, open brook in settlement E of Goderdzi Pass, 1850 m a.s.l., 41°38'03.4"N 42°32'48.6"E

G 04, Adjara, steep brook in spruce forest E of Goderdzi Pass, 1790 m a.s.l., 41°38'00.0"N 42°33'28.4"E

G 05, Adjara, Takidzeebi, Chvanis Tskali Stream 445 m a.s.l., 41°39'31.0"N 42°08'13.9"E

G 06, Guria region, snow-covered spring and brooks, tributary of Bzhuzha River, below Gomismta, 1910-1980 m a.s.l., 41°49'57"N 42°09'21"E

G 07, Imereti region, brook and spring, north slope of Zekari Pass, Kershaveti tributary, 2050 m a.s.l., 41°50'07"N 42°50'12"E

G 08, Kakheti region, Alazani River above the bridge of road 70, 350 m a.s.l., 41°59'30.6"N 45°34'40.5"E

G 09, Kakheti region, Batsara Nature Reserve, Batsara River and its sidebrook, 810 m a.s.l., 42°13'22.3"N 45°18'07.3"E (Fig. 9)

G 10, Kakheti region, David Gareja Monastery Complex, 465 m a.s.l., 41°30'11.0"N 46°05'59.5"E

G 11, Kakheti region, Stori forest brook along the road towards Abano Pass, 1215 m a.s.l., 42°13'01.7"N 45°28'32.4"E

G 12, Kakheti region, Gombori Pass, NW of the pass, spring and muddy brook, 1560 m a.s.l., 41°52'20"N 45°16'8"E

G 13, Kakheti region, Ilto River, above (N of) the Chartala village, 790 m a.s.l., 42°8'18"N 45°7'32"E

G 14, Kakheti region, Khadori gorge, Kvachadala forest brook, 1040 m a.s.l., 42°16'07.9"N 45°20'44.9"E

G 15, Kakheti region, Khadori gorge, forest torrent, 1090 m a.s.l., 42°16'21.3"N 45°20'57.1"E

G 16, Kakheti region, Khadori gorge, Samkura River, 885 m a.s.l., 42°15'10.8"N 45°19'55.1"E

G 17, Kakheti region, Kisiskhevi, Kisiskhevi River, channel and ruderal grassland, 560 m a.s.l., 41°54'13.4"N 45°33'51.1"E

G 18, Kakheti region, Napareuli, Lopota Lake and its inlet brook, 475 m a.s.l., 42°03'24.4"N 45°31'38.2"E

G 19, Kakheti region, Telavi, Bucha’s Guest House garden, 655 m a.s.l., 41°55'04.4"N 45°29'23.2"E

G 20, Kakheti, Stori River, 573 m a.s.l., 42°09'08.1"N 45°25'01.7"E

G 21, Kakheti, Stori River, 629 m a.s.l., 42°10'27.1"N 45°25'54.5"E

G 22, Kvemo Kartli region, Aiazmi, Zhamindzori Stream above (S of) the village, 1755 m a.s.l., 41°33'34.7"N 43°54'16.9"E

G 23, Kvemo Kartli region, Tsurtavi (Kolakiri), Khrami River and channel, littoral forest, 420 m a.s.l., 41°28'49.7"N 44°41'40.3"E

G 24, Kvemo Kartli region, Poladauri (Chatakh), Lokistskali Stream and springs, 700 m a.s.l., 41°19'50.9"N 44°29'52.9"E

G 25, Kvemo Kartli region, Poladauri (Samtsevrisi), Bolnisistskali (Poladauri) River, 665 m a.s.l., 41°20'17.2"N 44°30'16.3"E

G 26, Kvemo Kartli region, Sakdrioni, Ktsia River above Tsalka Reservoir, 1520 m a.s.l., 41°35'33.5"N 43°56'55.0"E

G 27, limit of Imereti and Samtskhe-Javakheti regions, Borjom-Kharagauli N. P., brook and spring, south slope of Zekari Pass, 2050 m a.s.l., 41°49'23''N 42°51'09"E

G 28, Mtskheta-Mtianeti region, Gveleti village, Gveleti Waterfalls, Tibistskali Stream, 1570 m a.s.l., 42°42'16"N 44°37'15"E

G 29, Mtskheta-Mtianeti region, above Juta, springs and brooklets in the Chaukhistskali Valley, near Fifth Season Hostel, 2340 m a.s.l., 42°34'28"N 44°45'15"E

G 30, Mtskheta-Mtianeti region, Gudauri, open brook along a military road, 2250 m a.s.l., 42°29'31.3"N 44°28'02.2"E

G 31, Mtskheta-Mtianeti region, Gudauri, temporary brook, 2045 m a.s.l., 42°28'01.0"N 44°28'48.5"E

G 32, Mtskheta-Mtianeti region, Gveleti, stream beneath Gveleti Small Waterfall, 1630 m a.s.l., 42°42'08.4"N 44°37'09.7"E

G 33, Mtskheta-Mtianeti region, Juta village, steep sidebrook, tributary of the Juta River, SSE slope, meadow, 2100 m a.s.l., 42°34'48"N 44°44'31"E

G 34, Mtskheta-Mtianeti region, Juta, Chaukhistskali River above (E of) the settlement, 2385 m a.s.l., 42°33'52.1"N 44°45'41.3"E

G 35, Mtskheta-Mtianeti region, Juta, spring brooks in the Chaukhistskali River valley, 2600 m a.s.l., 42°33'13.6"N 44°46'22.0"E

G 36, Mtskheta-Mtianeti region, Kharkheti (Nadibani), Aragvi River and its sidestream, 1235 m a.s.l., 42°24'57.7"N 44°36'15.2"E

G 37, Mtskheta-Mtianeti region, Kvemo Mleta, side spring and brook after bridge above the village, tributary of Tetri Aragvi River, 1490 m a.s.l., 42°25'55"N 44°30'27"E

G 38, Mtskheta-Mtianeti region, Kvemo Mleta, spring and its outlet, 1485 m a.s.l., 42°25'54.4"N 44°30'27.6"E

G 39, Mtskheta-Mtianeti region, Kvemo Mleta, spring and its outlet along a military road, 1485 m a.s.l., 42°25'54.3"N 44°30'27.2"E

G 40, Mtskheta-Mtianeti region, Mejilaurni, forest and bushy springs and outlets, 1270 m a.s.l., 42°19'25.4"N 44°38'43.9"E

G 41, Mtskheta-Mtianeti region, Meneso Spring, its outlet and surrounding bush, 940 m a.s.l., 42°14'48.5"N 44°40'29.2"E

G 42, Mtskheta-Mtianeti region, sidebrook of Chkheri River crossing forest road, 2050 m a.s.l., 42°40'12"N 44°36'37"E

G 43, Mtskheta-Mtianeti region, sidebrook of the Chaukhistskali Stream, 2700 m a.s.l., 42°33'25.1"N 44°46'56.9"E

G 44 Mtskheta-Mtianeti region, sidespring of Chkheri River beneath Altihut no. 3014 mountain shelter 2940 m a.s.l., 42°39'35.8"N 44°33'47.8"E

G 45 Mtskheta-Mtianeti region, sidespring of Chkheri River by Altihut 3014 mountain shelter 2960 m a.s.l., 42°39'32.0"N 44°33'39.9"E

G 46 Mtskheta-Mtianeti region, springs and brooklets in the High Juta Valley, above the small cascade, 3050 m a.s.l., 42°33'49"N 44°49'05"E

G 47, Mtskheta-Mtianeti region, springs and their outlet brooks N of Jvari Pass, 2380 m a.s.l., 42°31'07.4"N 44°27'52.3"E

G 48, Mtskheta-Mtianeti region, Tsinamkhari (Mejilaurni), stream and swampy sidebrook, 1180 m a.s.l., 42°19'28.7"N 44°38'55.1"E

G 49, Mtskheta-Mtianeti region, Tsinamkhari, forest edge swamp, 1150 m a.s.l., 42°19'26.5"N 44°39'11.8"E

G 50, Mtskheta-Mtianeti region, Tsinamkhari, forest stream, 1165 m a.s.l., 42°19'29.9"N 44°39'08.1"E

G 51, Mtskheta-Mtianeti region, Ukanamkhari, torrent, 1565 m a.s.l., 42°19'55.3"N 44°36'26.2"E

G 52, Mtskheta-Mtianeti region, upper section of Chaukhistskali Stream and its sideseep, 2645 m a.s.l., 42°33'19.2"N 44°46'37.5"E

G 53, Mtskheta-Mtianeti region, Zemo Mleta, brook and seeps along the military road, 1565 m a.s.l., 42°26'10.6"N 44°29'41.0"E

G 54, Mtskheta-Mtianeti region, Zemo Mleta, waterfall and seeps, 1565 m a.s.l., 42°26'10.2"N 44°29'41.9"E

G 55, Samtskhe-Javakheti region, spring and brooklet in grassy land, tributary of Borjomula River, above Bakuriani, 2270-2350 m a.s.l., 41°41'35"N 43°31'02"E

G 56, Tbilisi region, Tbilisi, Kura River shore by Hotel Rose, 380 m a.s.l., 41°39'36.2"N 44°53'29.8"E
